# Repeated Heat Regeneration of Bone Char for Sustainable Use in Fluoride Removal from Drinking Water

**DOI:** 10.3390/healthcare6040143

**Published:** 2018-12-08

**Authors:** H. M. Ayala S. Herath, Tomonori Kawakami, Masamoto Tafu

**Affiliations:** 1 Department of Environmental Engineering, Faculty of Engineering, Toyama Prefectural University, Toyama 939-0398, Japan; kawakami@pu-toyama.ac.jp; 2 National Institute of Technology, Toyama College, Toyama 939-8630, Japan; tafu@nc-toyama.ac.jp

**Keywords:** adsorption capacity, defluoridation, repeated heat regeneration, hydroxyapatite, ion exchange, chemical precipitation

## Abstract

The effectiveness of regenerated chicken bone char (CBC) in fluoride removal was investigated in the present study. Heat treatment was studied as the regeneration method. Results revealed that the CBC regenerated at 673 K yielded the highest fluoride adsorption capacity, hence, 673 K was the best regenerating temperature. The study continued up to five regeneration cycles at the best regenerating temperature; 673 K. The CBC accounted to 16.1 mg F/g CBC as the total adsorption capacity after five regeneration cycles. The recovery percentage of CBC reduced from 79% at the first regeneration to 4% after five regeneration cycles. The hydroxyapatite structure of CBC was not changed during the fluoride adsorption by five regeneration cycles. The ion exchange incorporated with the chemical precipitation occurred during the fluoride adsorption. The repeated regeneration of CBC is possible and it could be used as a low cost defluoridation technique to minimize the wastage of bone char.

## 1. Introduction

The contamination of groundwater by fluoride is one of the major global issues. An excess amount of fluoride intake causes dental fluorosis and skeletal fluorosis in humans [1]. Further, fluoride is supposed to be a suspicious cause for the multifactorial, deadly disease [2]; chronic kidney disease of unknown etiology (CKDu) in Sri Lanka [3]. The world health organization (WHO) guideline for fluoride in drinking water is 1.5 mg/L [4]. A high concentration of fluoride up to 30 mg/L was found in the groundwater of many countries in the world including Sri Lanka [3], India, Pakistan, China, West Indies, Spain, Holland, Poland, Italy, Iraq, Iran, Sudan, Uganda, Kenya, United Republic of Tanzania, Mexico, Thailand, Serbia, Ethiopia, Eritrea (North East Africa), West Africa, Southern Africa and North and South American countries [5]. Therefore, Defluoridation of drinking water is essential to protect the human life from fluoride contaminated water [6].

Adsorption is one of the techniques widely used as a low cost method in defluoridation [6]. Several adsorbents were reported in the literature such as activated carbon, activated alumina [7], clay, zeolites, fly ash, specific ion exchange resins [8], brick powder, bentonite, montmorillonite, laterite [9], rare earth oxides, red mud, hydroxyapatite, fluorspar, calcite, quartz, bauxite and gypsum [10]. Among the adsorbents, bone char was found to be a low cost, efficient [6] and environmentally friendly adsorbent.

Continuous usage of bone char in fluoride removal causes exhaustion of the bone char. Regeneration of exhausted bone char by heating is a new approach in fluoride removal [11].

In this study our attention was focused on the repeated regeneration of exhausted bone char in relation to its adsorption capacity, in order to reuse bone char in fluoride removal from contaminated water. 

## 2. Materials and Methods

### 2.1. Preparation of Chicken Bone Char 

The raw chicken bones were placed in closed metal containers and carbonized by an electrical muffle furnace (Koyo thermo system Co. Ltd. Tokyo, Japan, KBF 794N1) for 1.5 h at 873 K under an anaerobic condition. The carbonized CBC was washed several times with clean water and dried at 378 K in an electrical oven for one night [12]. 

### 2.2. Preparation of Fluoride Exhausted Bone Char 

**Figure 1:**
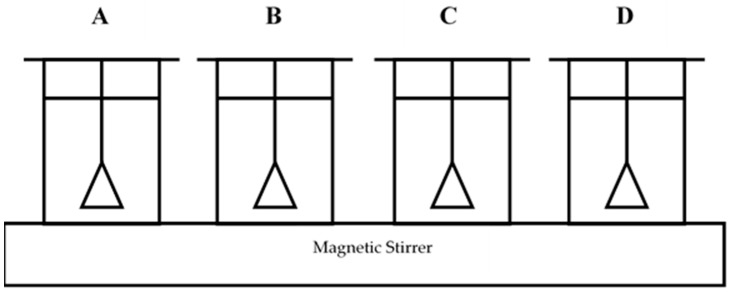
Schematic diagram of the adsorption setup used to prepare exhausted CBC.

The CBC with a diameter of 1–2 cm was used to prepare the exhausted bone char. Figure 1 shows the schematic diagram of the adsorption setup used to prepare exhausted bone char. Four setups (A, B, C and D) were used separately to prepare fluoride exhausted CBC for use in the same setup in the same manner for fluoride adsorption after the regeneration of CBC. 

Four liters (4 L) of synthesized drinking water with an initial fluoride concentration of 50 mg/L was prepared by dissolving sodium fluoride (NaF) in tap water. 1 L of the prepared solution was put into each 1 L beaker in each setup. Four tea bags each filled with ten grams of CBC were dipped in the solutions in the four setups. The four setups were allowed to adsorb fluoride for 25 days on a magnetic stirrer with a 270 rpm. The fluoride concentrations of the solutions were measured regularly using an ion selective electrode (ORION STAR A324 pH/ISE Meter and ORION 9609BNWP Ionplus Sure-Flow Fluoride Electrode). The Total Ionic Strength Adjustment Buffer (TISAB III) was added to each sample before analysis to avoid interference during the measurements. 

### 2.3. Regeneration of Chicken Bone Char 

Heat regeneration was selected as the regeneration method due to its easy operation and cost effectiveness. Since heat regeneration of CBC is not a chemical treatment method, the release of chemical components into treated water during a chemical regeneration may not occur. Therefore, the treated water may be safer for human consumption than from a chemical treatment.

The exhausted CBC in setups B, C and D was taken out from the tea bags after 25 days and dried at 338 K in an electrical oven for 24 h. Thereafter the CBC in the three setups (B, C and D) was reactivated by heat at 673 K, 773 K and 873 K under an anaerobic condition in closed metal containers in an electrical muffle furnace for 2 h [12,13]. The same amount of CBC used to prepare exhausted CBC in setups B, C and D was used for the regeneration (1st regeneration). The setup A was continued as the control setup without regeneration. In Figure 1, the A, B, C and D setups show the control, 673 K regenerated, 773 K regenerated and 873 K regenerated setups, respectively after regeneration. 

The CBC in setup B only (673 K regenerated CBC) was heat regenerated at 673 K under an anaerobic condition in a closed metal container in an electrical muffle furnace for 2 h 5 times repeatedly as the 1st, 2nd, 3rd, 4th and 5th regenerations. The setup A was continued as the control setup without regeneration, until the 5th regeneration was completed for setup B.

### 2.4. Fluoride Removal by Regenerated Bone Char

Regenerated CBC in setups B, C and D was returned to the same setups to continue the experiment after regeneration. The same amount of CBC used to prepare exhausted CBC in setups B, C and D was used in the adsorption setups after regeneration. 

Regenerated CBC in setup B was returned to the same setup to continue the experiment in the same manner after the 2nd, 3rd, 4th and 5th regenerations. The same amount of CBC used in the adsorption setup B in the 1st regeneration was used in the adsorption setup B after the 2nd, 3rd, 4th and 5th regenerations. The setup A was continued as the control setup without regeneration until the 5th regeneration.

Fluoride concentrations of the solutions were measured regularly by the ion selective electrode. The pH of the solutions was measured using a pH meter (ORION STAR A324 pH/ISE Meter and Beckman Electrode 511070). 

The F^−^ content in CBC before (raw CBC) and after the adsorption (control, 673 K regenerated CBC, 773 K regenerated CBC and 873 K regenerated CBC) was determined by digesting 0.1 g of CBC with 1 mL of concentrated nitric acid [12] [13]. F^−^ content in digested CBC was measured using the ion selective electrode. The X-ray diffraction (XRD) of hydroxyapatite (HAP) and CBC before and after the adsorption were analyzed using MiniFlex (Rigaku Co., Tokyo, Japan); X-ray 30 kV/15 mA, radiation CuK alpha line (Ni filter), scintillation detector. 

Anion and cation concentrations of the solutions were measured using ion chromatography (for anions: Dionex ICS-2000, separation column IonPac AS18, eluent KOH 23–40 mmol/L (gradient), suppressor ASRS 300 4 mm; for cations: Dionex ICS-1500, separation column IonPac CS12, eluent methanesulfonic acid 30 mmol/L (isocratic), suppressor CSRS 500 4 mm). 

## 3. Results and Discussion

### 3.1. Preparation of Fluoride Exhausted Bone Char 

**Figure 2:**
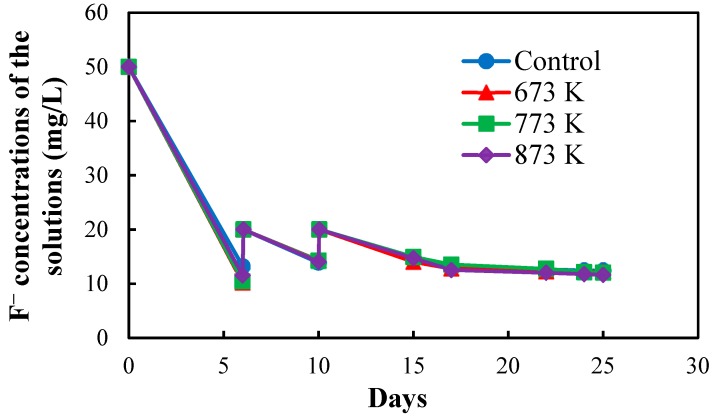
Fluoride concentrations of the solutions in the four setups used to prepare exhausted CBC.

Figure 2 shows the fluoride concentrations of the solutions in the four setups used to prepare exhausted CBC throughout the operation period of 25 days. The fluoride concentrations of the solutions in the four setups were increased to 20 mg/L at days 6 and 10 to allow them to adsorb more amount of fluoride in order to obtain a quick equilibrium with the F^−^ in the solutions. NaF solution with a concentration of 1000 mg/L was used to increase the fluoride concentrations of the solutions in the four setups. 

### 3.2. Fluoride Removal by Regenerated Bone Char

**Figure 3:**
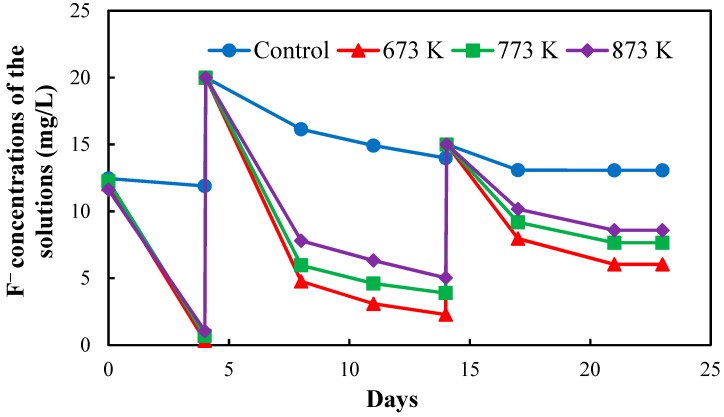
Fluoride concentrations of the solutions in the four setups after 1st regeneration of CBC.

Figure 3 shows the fluoride concentrations of the solutions in the four setups after the 1^st^ regeneration of CBC throughout the operation period of 23 days. The fluoride concentrations of the solutions in the four setups were increased to 20 mg/L at day 4 and 15 mg/L at day 14 to obtain a quick equilibrium with the fluoride in the solutions. NaF solution with the concentration of 1000 mg/L was used to increase the fluoride concentrations of the solutions in the four setups. 

The equilibrium fluoride concentrations of the solutions in the four setups were used to determine the fluoride adsorption capacity of regenerated CBC. 

**Figure 4:**
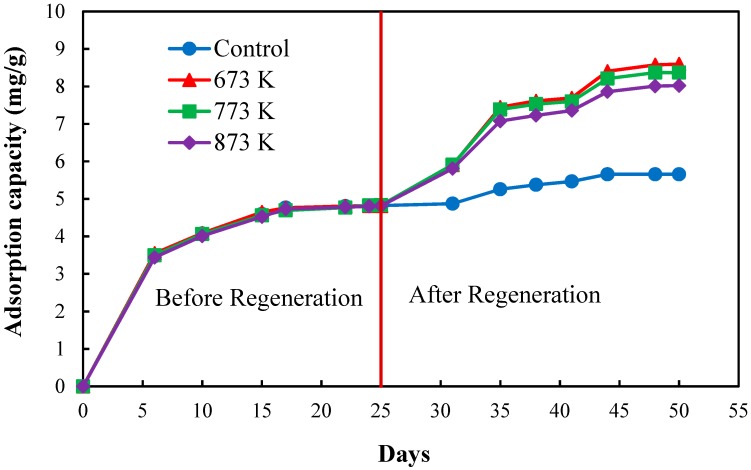
Adsorption capacities of CBC used for the 1st regeneration obtained from the mass balance calculation for the solutions throughout the operation period.

Figure 4 shows the effect of the 1st regeneration on the adsorption capacities of CBC obtained from the mass balance calculation for the solutions throughout the operation period of 50 days. The total adsorption capacities both before and after the regeneration were considered in order to evaluate the adsorption capacities after each regeneration cycle of CBC [12]. 

The CBC regenerated at 673 K showed the highest total adsorption capacity; hence, 673 K was the best regenerating temperature. The total adsorption capacity of CBC regenerated at 773 K was slightly lower than that of the CBC regenerated at 673 K but was slightly higher than that of the CBC regenerated at 873 K. The adsorption capacity of regenerated CBC decreased with increasing temperature. When comparing the total adsorption capacities of regenerated CBC and control CBC, the total adsorption capacities of regenerated CBC were higher than that of the control CBC confirming that the heat regeneration of CBC was an effective method in defluoridation of drinking water. 

Adsorption of fluoride onto heat regenerated bone char has been reported in the literature. Kaseva studied heat regenerated cattle bone char in fluoride removal at different temperatures and determined that the best regenerating temperature was 773 K [11]. Nigri et al. studied heat regenerated bovine bone char in fluoride removal at different temperatures and determined that the best regenerating temperature was 673 K [14], as we obtained in our study. 

**Figure 5:**
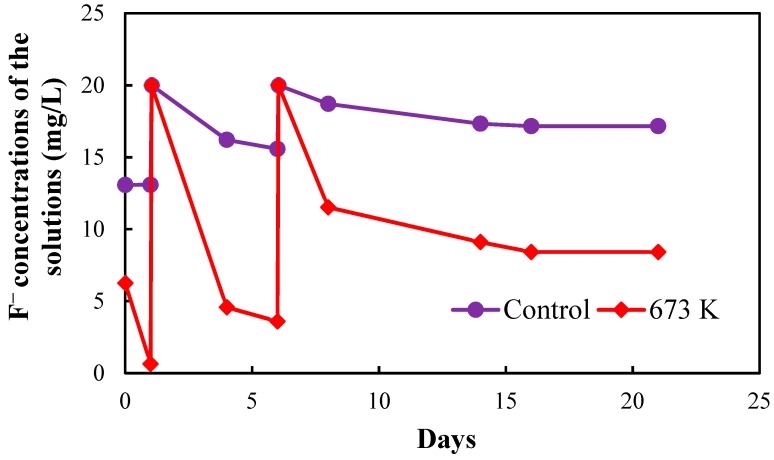
Fluoride concentrations of the solutions in the two setups after 2nd regeneration of CBC.

**Figure 6:**
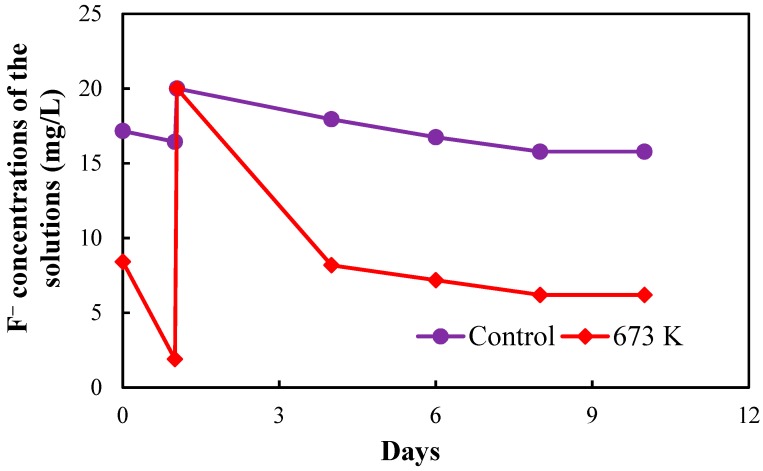
Fluoride concentrations of the solutions in the two setups after 3rd regeneration of CBC.

**Figure 7:**
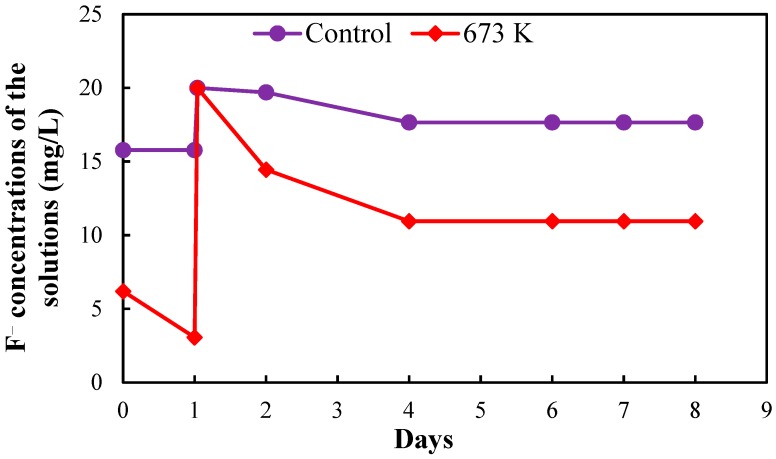
Fluoride concentrations of the solutions in the two setups after 4th regeneration of CBC.

**Figure 8:**
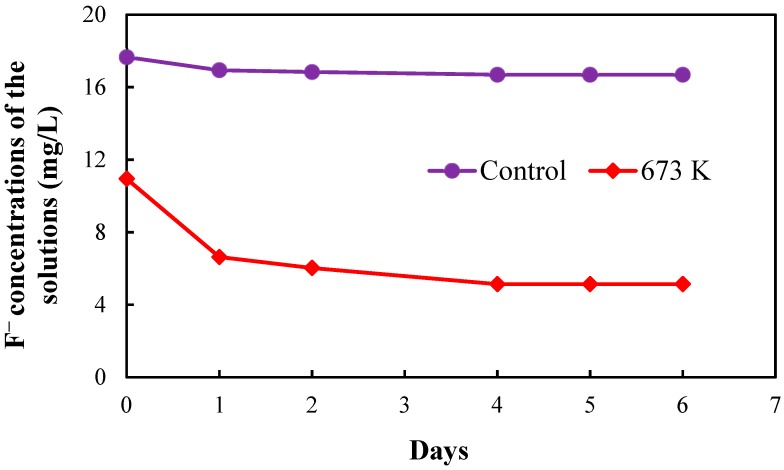
Fluoride concentrations of the solutions in the two setups after 5th regeneration of CBC.

Figure 5, Figure 6, Figure 7 and Figure 8, respectively, show the fluoride concentrations of the solutions; control and 673 K regenerated setups after the 2nd, 3rd, 4th and 5th regenerations of CBC throughout the operation periods of 21, 10, 8 and 6 days, respectively. 

The operation period of the 2nd regeneration was 21 days. The fluoride concentrations of the solutions in the two setups were increased to 20 mg/L at day 1 and day 6 in the 2nd regeneration. The operation period of the 3rd regeneration was 10 days. The fluoride concentrations of the solutions in the two setups were increased to 20 mg/L at day 1 in the 3rd regeneration. The operation period of the 4th regeneration was 8 days. The fluoride concentrations of the solutions in the two setups were increased to 20 mg/L at day 1 in the 4th regeneration. The operation periods of the 5th regeneration was 6 days. The fluoride concentrations of the solutions in the two setups were not increased in the 5th regeneration.

NaF solution with the concentration of 1000 mg/L was used to increase the fluoride concentrations of the solutions in the two setups. The fluoride concentrations of the two setups were increased in the 2nd, 3rd and 4th regenerations to obtain a quick equilibrium with the fluoride in the solutions.

The equilibrium fluoride concentrations of the solutions in the two setups were used to determine the fluoride adsorption capacity of CBC after the 2nd, 3rd, 4th and 5th regenerations. 

**Figure 9:**
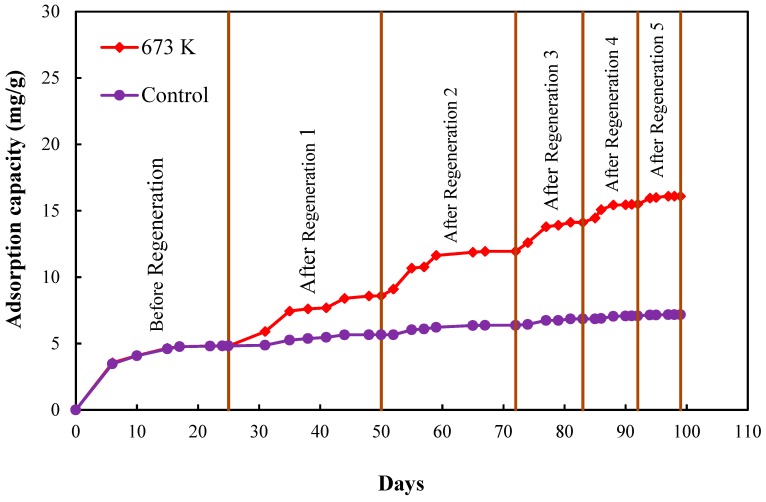
Adsorption of F^−^ by CBC (control and 673 K regenerated CBC) before and after 1st, 2nd, 3rd, 4th and 5th regenerations obtained from the mass balance calculation for the solutions throughout the operation period.

Figure 9 shows the F^−^ adsorbed by CBC before and after the 1st, 2nd, 3rd, 4th and 5th regenerations, obtained from the mass balance calculation for the solutions throughout the operation period of 99 days for the control and the 673 K regenerated setups. Table 1 shows the total amount of F^−^ adsorbed by CBC before and after regeneration, obtained from the mass balance calculation for the solutions; control, 673 K regenerated, 773 K regenerated and 873 K regenerated setups. Table 2 shows the adsorption capacities of CBC before and after regeneration obtained by the acid digestion of CBC; control, 673 K regenerated, 773 K regenerated and 873 K regenerated setups. F^−^ content in CBC before the adsorption (raw CBC) was considered when calculating the adsorption capacity by acid digestion of CBC. 

Adsorption capacities of CBC obtained from the mass balance calculation for the solutions were coincided with the adsorption capacities obtained from the acid digestion of CBC. However, there is a slight difference between the adsorption capacities obtained from the mass balance calculation for the solutions and from the acid digestion. This may be due to an error in the volume measurement when sampling the solutions for the regular measurement of F^−^ concentration of the solution in each setup.

Table 3 shows the recovery rate of 673 K regenerated CBC during the five regeneration cycles. The recovery rate of CBC gradually decreased after each regeneration cycle. The recovery rate decreased approximately by half with each regeneration cycle. It is possible to reuse CBC five times repeatedly by continuous heat regeneration. This phenomenon is environmentally friendly, since it minimizes the wastage of CBC. Repeated heat regeneration of CBC is a new approach in defluoridation techniques. No study has used bone char continuously for five heat regeneration cycles in fluoride removal. 

**Figure 10:**
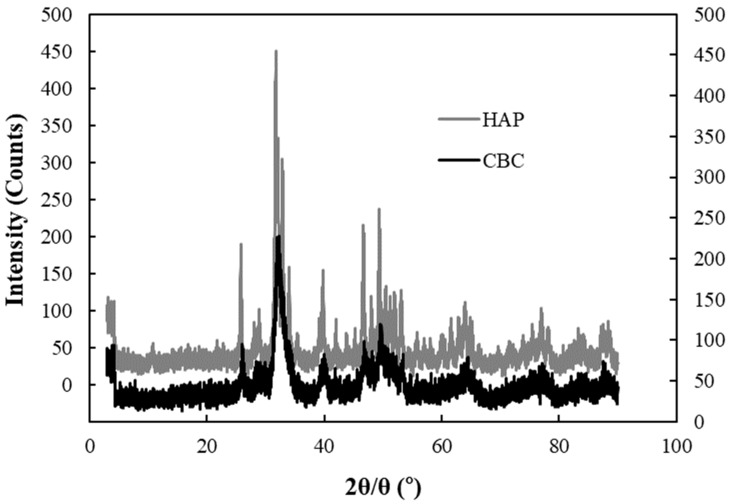
XRD patterns of CBC and HAP. HAP (left Y axis), CBC (right Y axis).

The major component of chicken bone char (CBC) is Hydroxyapatite (HAP) [6,12]. Figure 10 shows the XRD patterns of HAP and CBC. The XRD patterns overlap, indicating that the major component of CBC is HAP. 

Fluoride removal by chicken bone char (CBC) is associated with the mechanism of ion exchange. 

The ion exchange mechanism is shown in Equation (1):

(1) $${\mathrm{Ca}}_{10}({\mathrm{PO}}_{4}{)}_{6}(\mathrm{OH}{)}_{2}\;+\;2\;F^{-}\to {\mathrm{Ca}}_{10}({\mathrm{PO}}_{4}{)}_{6}F_{2}\;↓+\;+\;2\;{\mathrm{OH}}^{-}\;$$

The hydroxyl ion in HAP in bone char (CBC) is replaced by fluoride ion, in the presence of fluoride ions. Insoluble fluorapatite (FAP) is formed from HAP [15] and the hydroxyl ion is released into the solution, as represented in Equation (1) [16].

The hydroxyl ion can replace fluoride ion in mineral structures because of the same charge and similar radius of F^−^ and OH^−^ [17].

**Figure 11:**
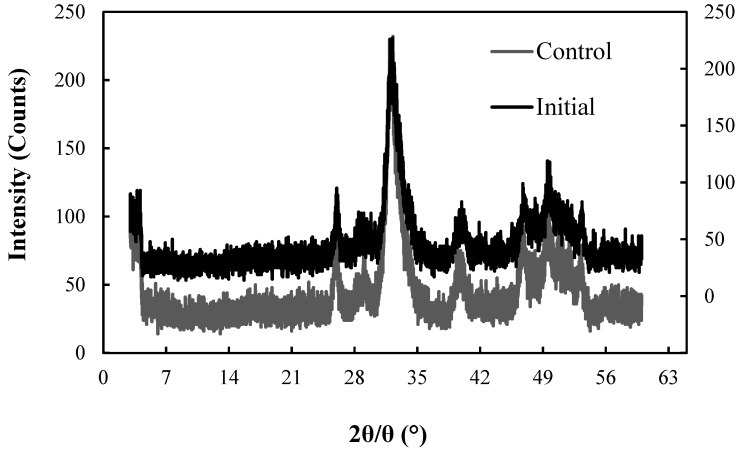
XRD patterns for the initial CBC and control setup after F^−^ adsorption. Initial (right Y axis), After 99 days’ adsorption for control setup (left Y axis).

**Figure 12:**
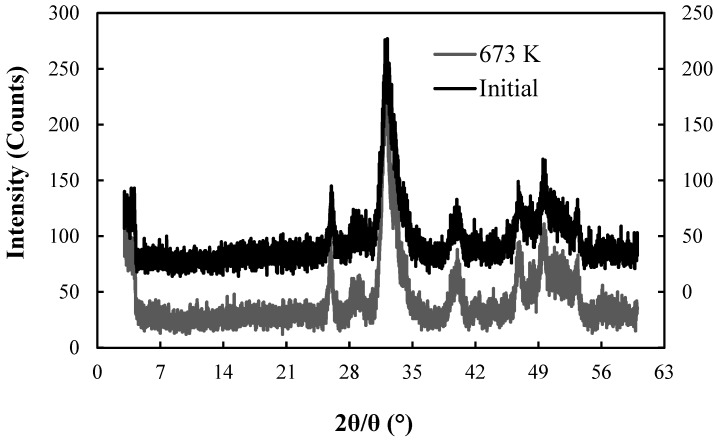
XRD patterns for the initial CBC and CBC after F^−^ adsorption. Initial (right Y axis), After 99 days’ adsorption for 673 K regenerated setup (left Y axis).

Figure 11 and Figure 12 show the XRD patterns of CBC before (initial) and after the fluoride adsorption for the control and the 673 K regenerated CBC, respectively. 

It was obvious that the XRD patterns of CBC before and after adsorption overlapped reveling that the structure of CBC has not changed during the fluoride adsorption by heat regenerated CBC, even after five regeneration cycles. The HAP structure of chicken bone char (CBC) was not changed by repeated heat regenerations. The fluoride incorporated to HAP (FAP) was detected by the acid digestion of CBC (Table 2) after 1st, 2nd, 3rd, 4th and 5th heat regenerations. The Equation (1) took place during the adsorption [12].

Nigri et al., showed that the XRD patterns of bone char were not changed during the fluoride adsorption by heat regenerated bone char by studying bovine bone char for a single heat regeneration cycle. They also reported the formation of FAP during the fluoride adsorption by heat regenerated bovine bone char [14]. 

Kaseva reported that the fluoride adsorbed on exhausted bone char (CBC) evaporated as HF gas during the heat regeneration, as shown in Equation (2) [11]:

(2) $${\mathrm{Ca}}_{10}({\mathrm{PO}}_{4}{)}_{6}F_{2}\;+\;2\;{\mathrm{OH}}^{-}\;\to {\mathrm{Ca}}_{10}({\mathrm{PO}}_{4}{)}_{6}\;+\;2\;\mathrm{HF}\;↑\;+\;O_{2}\;↑$$

The same amount of adsorbed fluoride onto CBC after the 1st, 2nd, 3rd, 4th and 5th regenerations (Table 1) was detected both by the acid digestion of CBC (Table 2) and by the mass balance calculation. It was concluded that the adsorbed fluoride during the formation of exhausted CBC was not evaporated as HF gas during the heat regeneration as mentioned in Equation (2) [12]. 

Nigri et al., reported that the diffusion of adsorbed fluoride inside to the bone char pores was observed during the heat regeneration [14]. This phenomenon might have occurred during our study as well. 

Table 4 shows the solution pH. There was an increase in pH in the final solutions after regeneration when compared to the initial solution. The pH value of the solution should increase due to the release of OH^−^ into the solution with the formation of FAP as shown in Equation (1). 

Table 5 shows the changes in anion and cation concentrations of the solutions used for the regeneration before and after adsorption. The Na^+^ in the final solutions increased mainly due to the addition of NaF to the solutions. A considerable increase of Na^+^ was detected in the regenerated solutions; 673 K, 773 K and 873 K when compared to the control. PO_4_^3−^ and K^+^ were dissolved from CBC [6] and increased their concentration in the final solutions. SO_4_^2−^ was also detected in the final solutions. A considerable increase of SO_4_^2−^ was detected in the regenerated solutions; 673 K, 773 K and 873 K when compared to the control. Mg^2+^ and Ca^2+^ ions were adsorbed by CBC and were not detected in the final solutions. 

Fluoride removal by bone char (CBC) is also associated with the precipitation of calcium fluoride (CaF_2_). 

The CaF_2_ precipitation mechanism is shown in Equation (3):

(3) $${\mathrm{Ca}}_{10}({\mathrm{PO}}_{4}{)}_{6}(\mathrm{OH}{)}_{2}\;+\;20\;F^{-}\;+\;2\;H^{+}\;\to \;10\;{\mathrm{CaF}}_{2}\;↓+\;6\;{{\mathrm{PO}}_{4}}^{3-}\;+\;2\;H_{2}O$$

HAP in bone char (CBC) precipitates into calcium fluoride (CaF_2_), in the presence of excess fluoride ions. Phosphate (PO_4_^3−^) in HAP is released into the solution [12] as shown in Equation (3) [17]. 

The Equation (3) also took place during the adsorption, since phosphate was detected in the final solutions before and after the 1st, 2nd, 3rd, 4th and 5th heat regenerations, as well as in the control setup [12]. 

According to the Equation (3), the molar ratio of F^−^ to PO_4_^3−^ in our study was 0.0938 mol/L:0.0011 mol/L (F^−^ = ((163.58 − 74.49) mg/L/1000/19 g/mol) × 20; based on adsorbed F^−^ for control (74.49 mg/L) and 673 K regenerated CBC after 5th regeneration (163.58 mg/L)), PO_4_^3−^ = ((56 − 38) mg/L/1000/94.97 g/mol) × 6; based on the PO_4_^3−^ data for final solutions for control and 673 K regenerated CBC after the 5th regeneration reported in Table 5). 

The molar ratio of F^−^ to PO_4_^3−^ obtained in our study did not coincide with the molar ratio of F^−^ to PO_4_^3−^ in Equation (3). The amount of F^−^ was much higher than the appropriate molar ratio representing by Equation (3). The molar ratio obtained by our study for F^−^ and PO_4_^3−^ was evidence that the adsorption of F^−^ was not only due to CaF_2_ precipitation. Therefore, we can confirm that the ion exchange incorporated with CaF_2_ precipitation occurred during the adsorption [12]. 

Nigri et al., detected phosphate in fluoride adsorption experiments by heat regenerated bovine bone char for a single regeneration cycle and showed that the chemical precipitation occurred during the fluoride adsorption by heat regenerated bone char [14]. 

Further, it is possible that the unopened pores in chicken bone char (CBC) may reactivate the fluoride adsorption [12] after each regeneration cycle, instead of the above mentioned phenomenon. 

## 4. Conclusions

Fluoride removal from contaminated water is essential in order to protect human health. CBC was selected as a low cost adsorbent for fluoride removal. Regeneration of CBC was investigated and heat regeneration was selected as the regeneration method. It was found that 673 K was the best regenerating temperature. Repeated heat regeneration of CBC was studied by five heat regeneration cycles at 673 K. The recovery percentage of CBC decreased gradually after each regeneration cycle; however, the total F^−^ adsorbed on to CBC was 16.1 mg/g, which was almost two times higher than that without regeneration. 

The XRD patterns of CBC before and after the five regeneration cycles revealed that the structure of CBC did not change even after five continuous regeneration cycles. The results revealed that the ion exchange incorporated with the CaF_2_ precipitation occurred during the adsorption. 

The repeated heat regeneration of CBC is possible and it is a new approach in fluoride removal techniques. CBC can be reused to minimize the wastage of CBC by using this technique, hence, it is a sustainable, environmentally friendly and efficient technique in fluoride removal. Therefore, this technique is highly recommended for developing countries. 

## Figures and Tables

**Table 1:** Adsorption capacities of CBC used for the regeneration obtained from the mass balance calculation for the solutions.

Setups	State	Weight of CBC (g)	Adsorbed F^−^ (mg)	Adsorption Capacity (mg/g)	Total Adsorption Capacity (mg/g)
Control	Before regeneration	10.07	48.57	4.82	7.17
	After regeneration 1		8.41	0.84	
	After regeneration 2		7.22	0.72	
	After regeneration 3		4.84	0.48	
	After regeneration 4		2.31	0.23	
	After regeneration 5		0.89	0.09	
673 K regenerated	Before regeneration	10.66	51.25	4.81	16.11
	After regeneration 1	9.87	37.39	3.79	
	After regeneration 2	9.81	32.88	3.35	
	After regeneration 3	8.99	19.62	2.18	
	After regeneration 4	8.75	12.00	1.37	
	After regeneration 5	8.60	5.22	0.61	
773 K regenerated	Before regeneration	10.21	49.35	4.83	8.37
	After regeneration	9.52	33.66	3.54	
873 K regenerated	Before regeneration	10.36	49.90	4.81	8.02
	After regeneration	9.62	30.86	3.21	

**Table 2:** Adsorption capacities of CBC according to the HNO_3_ digestion.

Setups	F^−^ Adsorption Capacity (mg/g)		
	F^−^ Content in CBC before Adsorption (raw CBC)	F^−^ Content in CBC after Adsorption	Net Adsorption
Control	0.20	6.84	6.64
673 K regenerated		15.77	15.57
773 K regenerated		7.93	7.73
873 K regenerated		7.63	7.43

**Table 3:** Recovery rate of 673 K regenerated CBC.

State	Adsorption Capacity (mg/g)	Recovery Rate (%)
Before regeneration	4.81	-
After regeneration 1	8.60	79
After regeneration 2	11.95	39
After regeneration 3	14.13	18
After regeneration 4	15.50	10
After regeneration 5	16.11	4

**Table 4:** Solution pH.

Solutions	State	pH
Initial solutions	-	7.39
Final solutions before regeneration	Control	8.23
	673 K	8.31
	773 K	8.28
	873 K	8.29
Final solutions after regeneration 1	Control	8.28
	673 K	8.21
	773 K	8.27
	873 K	8.47
Final solution after regeneration 2	Control	8.31
	673 K	8.23
Final solution after regeneration 3	Control	8.27
	673 K	8.23
Final solution after regeneration 4	Control	8.27
	673 K	8.31
Final solution after regeneration 5	Control	8.26
	673 K	8.35

**Table 5:** Anion and cation concentrations of solutions.

Solutions	Concentrations (mg/L)										
Type	State	F^−^	Cl^−^	SO_4_^2^^−^	NO_3_^−^	PO_4_^3^^−^	Na^+^	NH_4_^+^	K^+^	Mg^2+^	Ca^2+^
Initial solutions	-	50	10	6	1	0	68	0	1	1	7
Final solutions before regeneration	Control	12	10	10	1	46	86	1	13	0	0
	673 K	12	11	12	1	49	89	1	15	0	0
	773 K	12	10	10	1	49	88	1	13	0	0
	873 K	12	13	12	1	45	87	1	14	0	0
Final solutions after regeneration 1	Control	13	9	9	1	43	85	1	12	0	0
	673 K	6	12	126	1	32	126	2	15	0	0
	773 K	8	11	90	1	45	116	2	13	0	0
	873 K	9	15	39	1	46	121	2	16	0	0
Final solution after regeneration 2	Control	17	8	8	1	42	88	1	11	0	0
	673 K	8	11	166	1	40	149	2	13	0	0
Final solution after regeneration 3	Control	16	7	7	1	40	83	1	10	0	0
	673 K	6	11	182	1	44	158	3	13	0	0
Final solution after regeneration 4	Control	18	7	7	1	38	82	1	9	0	0
	673 K	11	11	173	3	52	174	3	13	0	0
Final solution after regeneration 5	Control	17	7	7	1	38	82	1	9	0	0
	673 K	5	11	181	5	56	176	3	13	0	0
